# Zinc Deficiency Among Patients With Inflammatory Bowel Disease: A Systematic Review and Meta-Analysis

**DOI:** 10.7759/cureus.102684

**Published:** 2026-01-31

**Authors:** Rahman Hameed Mohammed Abdul, Nafisa Reyaz, Abebe Yigzaw, Bilal Asad Mohammed, Helai Hussaini, Adil Amin, Mohammed Qasim Rauf, Neelum Ali

**Affiliations:** 1 Gastroenterology and Hepatology, Royal Derby Hospital, Derby, GBR; 2 Medicine, Jawaharlal Nehru Medical College and Hospital, Aligarh, IND; 3 Internal Medicine, Addis Ababa University School of Medicine, Addis Ababa, ETH; 4 General Practice, Government Medical College, Siddipet, Hyderabad, IND; 5 Internal Medicine, West Anaheim Medical Center, Anaheim, USA; 6 Cardiology, PNS (Pakistan Navy Ship) Shifa, Karachi, PAK; 7 Trauma and Orthopaedics, The Hillingdon Hospitals NHS Foundation Trust, London, GBR; 8 Internal Medicine, University of Health Sciences, Lahore, PAK

**Keywords:** crohn's disease, inflammatory bowel disease, meta-analysis, ulcerative colitis, zinc deficiency

## Abstract

This systematic review and meta-analysis aimed to comprehensively evaluate the prevalence of zinc deficiency in patients with inflammatory bowel disease (IBD). A systematic literature search was conducted across PubMed/MEDLINE (Medical Literature Analysis and Retrieval System Online), Embase, Scopus, Web of Science, and Cochrane Library from inception to November 2025. Studies reporting zinc deficiency prevalence in IBD patients using validated biomarkers were included. Two independent reviewers performed study selection, data extraction, and quality assessment using the Newcastle-Ottawa Scale. Meta-analysis was performed using a random-effects model, with subgroup analyses conducted by IBD subtype. Seven studies comprising 2,403 IBD patients were included. The pooled prevalence of zinc deficiency was 35% (95% CI: 19-52%), with substantial heterogeneity (I² = 98.5%). Subgroup analysis revealed a higher prevalence in Crohn's disease patients (40%, 95% CI: 21-59%) compared to ulcerative colitis patients (33%, 95% CI: 18-51%). These findings underscore the significant burden of zinc deficiency in IBD populations and highlight the need for routine nutritional screening, particularly in Crohn's disease patients. Clinicians should consider zinc assessment as part of comprehensive IBD management, with targeted supplementation for deficient patients to potentially improve clinical outcomes and quality of life.

## Introduction and background

Inflammatory bowel disease (IBD), which includes Crohn’s disease (CD) and ulcerative colitis (UC), comprises a group of chronic inflammatory conditions of the gastrointestinal tract characterized by a relapsing-remitting course, with a global prevalence estimated at 0.3% in Western populations and rising incidence in newly industrialized countries [1,2]. The multifactorial etiology of IBD involves complex interactions between genetic susceptibility, environmental factors, gut microbiota dysbiosis, and immune dysregulation, leading to chronic intestinal inflammation and progressive bowel damage [3]. Beyond the primary gastrointestinal manifestations, patients with IBD frequently experience a wide spectrum of nutritional deficiencies that significantly impact disease outcomes, quality of life, and treatment responses [4].

As an essential trace element, zinc is involved in over 300 enzymatic reactions and is crucial for immune regulation, protein synthesis, wound healing, DNA synthesis, and cellular division [5]. The human body cannot synthesize or store zinc efficiently, necessitating regular dietary intake to maintain adequate physiological levels [6]. Zinc deficiency has been associated with impaired immune responses, increased oxidative stress, compromised intestinal barrier function, and delayed wound healing-all of which are particularly relevant in the pathophysiology and management of IBD [7,8]. Despite its clinical significance, zinc deficiency often remains underdiagnosed in IBD populations due to non-specific symptoms and challenges in the accurate measurement of zinc status [9].

Multiple mechanisms contribute to zinc deficiency in IBD patients, including reduced dietary intake due to food aversions and restrictive diets, malabsorption secondary to intestinal inflammation and mucosal damage, increased gastrointestinal losses through diarrhea and protein-losing enteropathy, and drug-nutrient interactions with medications such as sulfasalazine and corticosteroids [10,11]. Furthermore, chronic inflammation itself increases zinc requirements through heightened metabolic demands and sequestration of zinc in inflammatory cells [12]. The prevalence of zinc deficiency in IBD has been reported variably across studies, ranging from 15% to 40% in pediatric populations and 40% to 50% in adult cohorts, though methodological heterogeneity limits definitive conclusions [13,14].

This study aims to comprehensively evaluate the prevalence of zinc deficiency in patients with IBD, examine potential sources of heterogeneity, and identify knowledge gaps that warrant future research to optimize nutritional management and clinical outcomes in this vulnerable population.

## Review

Methodology

Literature Search and Search Strategy

An extensive and systematic search of the literature was carried out using several electronic databases, namely PubMed/MEDLINE (Medical Literature Analysis and Retrieval System Online), Embase, Scopus, Web of Science, and the Cochrane Library, covering all records available from database inception up to November 15, 2025. The search strategy was designed collaboratively with a professional medical librarian and incorporated both controlled vocabulary terms (such as Medical Subject Headings (MeSH)) and unrestricted text words. These terms were structured around three core domains: (i) inflammatory bowel diseases, including CD and UC, (ii) zinc-related outcomes, encompassing zinc deficiency, hypozincemia, zinc levels, and trace elements, and (iii) measures of disease burden, such as prevalence and epidemiological frequency.

For PubMed, an example of the search syntax was as follows: (“inflammatory bowel disease” OR “IBD” OR “Crohn’s disease” OR “ulcerative colitis”) AND (“zinc” OR “zinc deficiency” OR “hypozincemia” OR “zinc status” OR “trace element*”) AND (“prevalence” OR “epidemiology” OR “frequency” OR “incidence”). This strategy was customized for each database in accordance with its unique indexing structure and search functionalities. To ensure comprehensive coverage, the reference lists of all eligible articles and relevant review papers were manually examined to identify additional studies. Searches of grey literature were also undertaken, including conference abstracts, clinical trial registries (ClinicalTrials.gov and the WHO International Clinical Trials Registry Platform), and dissertation repositories. No restrictions on language were imposed, and studies published in languages other than English were translated as required. The search process was independently conducted by two reviewers, and any discrepancies were resolved through consensus-based discussion.

Eligibility Criteria and Study Selection

Studies were considered eligible if they satisfied the following conditions: first, the study population consisted of individuals diagnosed with inflammatory bowel disease, including CD, UC, or IBD-unclassified, with diagnoses established using recognized clinical, endoscopic, radiological, and histopathological standards; second, the study provided estimates of zinc deficiency prevalence or presented adequate information to allow calculation of prevalence; third, zinc deficiency was assessed using serum or plasma zinc levels or other validated indicators, with explicitly defined threshold values; and fourth, the investigation employed an observational design, such as cross-sectional, cohort, or case-control studies.

Studies were excluded if they met any of the following criteria: publications in the form of case reports, small case series involving fewer than 10 participants, editorials, letters, commentaries, or narrative reviews; studies conducted exclusively in healthy individuals or populations without inflammatory bowel disease; studies that focused solely on the effects of zinc supplementation without reporting baseline prevalence data; or multiple reports derived from the same study population, in which case only the most complete or relevant dataset was retained.

All records identified through the literature search underwent initial title and abstract screening by two reviewers working independently, using predefined inclusion and exclusion criteria. Articles deemed potentially relevant were retrieved in full text and reassessed independently for eligibility. Any discrepancies between reviewers were resolved through discussion, and when consensus could not be reached, adjudication was performed by a third senior reviewer. The overall study selection procedure was summarized and reported in accordance with the Preferred Reporting Items for Systematic Reviews and Meta-Analyses (PRISMA) guidelines [15].

Data Extraction

A standardized data extraction form was developed using Microsoft Excel (Microsoft Corporation, Redmond, Washington, United States). Two independent reviewers extracted data from all included studies, with discrepancies resolved through consensus or third-party adjudication. The following information was extracted: (i) study characteristics: first author, publication year, country, study design, and sample size, (ii) population characteristics: age, sex distribution, IBD type (CD, UC, IBD-unclassified), and disease duration, and (iii) outcome data: number of patients with zinc deficiency, total number of patients assessed.

Quality Assessment

Two reviewers independently evaluated the methodological quality of the included observational studies using the Newcastle-Ottawa Scale (NOS), a validated tool for assessing non-randomized studies in systematic reviews and meta-analyses [16]. The NOS assigns stars across three core categories: (i) selection of study participants, including sample representativeness, sample size, response rate, and method of exposure measurement, (ii) comparability of study groups, focusing on adjustment for key confounders, and (iii) outcome evaluation, which covers outcome measurement and the appropriateness of statistical analyses.

Data Analysis

All statistical analyses were carried out in RStudio (R Foundation for Statistical Computing, Vienna, Austria). Overall pooled prevalence and the corresponding 95% confidence intervals (CIs) were derived using a random-effects model, which incorporates both within-study and between-study variability. Between-study heterogeneity was evaluated using Cochran’s Q test (with p<0.10 considered evidence of significant heterogeneity) and quantified using the I² statistic, where values above 75% reflected high heterogeneity, 50-75% represented moderate heterogeneity, and values below 50% indicated low heterogeneity. To investigate potential contributors to heterogeneity, subgroup analyses were performed according to inflammatory bowel disease subtype (CD vs. UC).

Results

**Figure 1:**
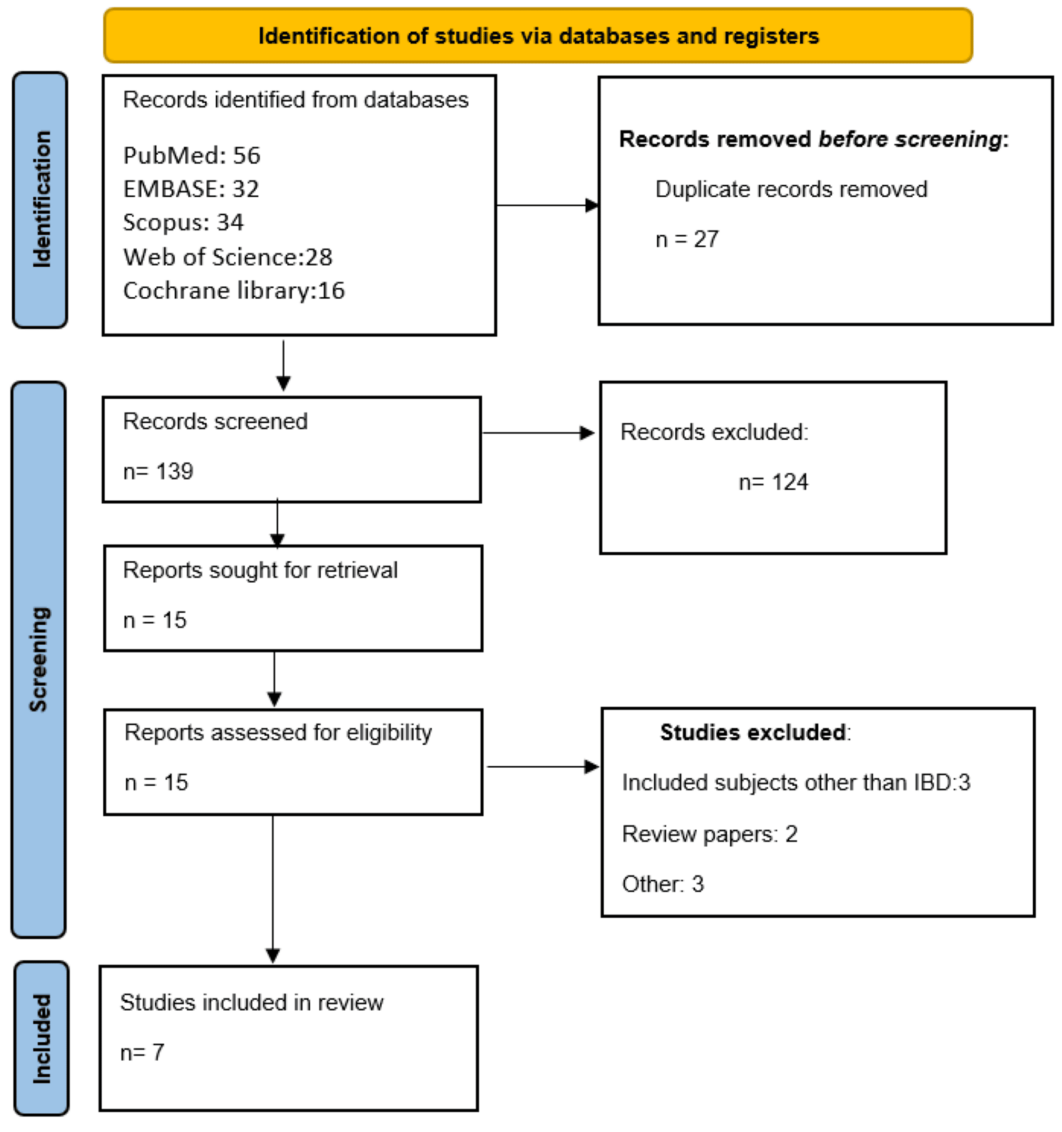
PRISMA flowchart PRISMA: Preferred Reporting Items for Systematic reviews and Meta-Analyses

The electronic database search identified a total of 166 records. Following the removal of duplicate entries, 139 articles underwent preliminary screening based on their titles and abstracts. From this stage, 15 studies were deemed potentially relevant and were subjected to detailed evaluation using the predefined inclusion and exclusion criteria. After full-text assessment, only seven studies fulfilled the eligibility requirements and were ultimately incorporated into the meta-analysis. The study selection process, including the number of records assessed at each stage, is depicted in the PRISMA flow diagram (Figure 1). Descriptive information for the included studies is summarized in Table 1.

**Table 1:** Study characteristics of included articles UC: ulcerative colitis; CD: Crohn's disease; NR: not reported

Study (Author, Year)	Study Design	Region	Sample Size	CD (n)	UC (n)	Age (Years)	Male (n)
Almuhaya et al., 2025 [17]	Retrospective	Saudi Arabia	530	354	86	29	243
Han et al., 2017 [18]	Retrospective	Korea	83	34	49	32	64
MacMaster et al., 2021 [19]	Prospective	United Kingdom	93	59	30	47.5	38
Sakurai et al., 2021 [20]	Retrospective	Japan	482	276	206	45	36
Schneider et al., 2020 [21]	Cross-sectional	Switzerland	154	98	56	41.4	75
Siva et al., 2018 [12]	Retrospective	United States	996	773	223	NR	468
Soltani et al., 2021 [22]	Retrospective	Iran	65	65	0	41	37

In total, seven studies were analyzed, encompassing retrospective, prospective, and cross-sectional study designs, and originating from a wide range of geographic locations, including Saudi Arabia, South Korea, the United Kingdom, Japan, Switzerland, the United States, and Iran. Study sample sizes showed considerable variation, with participant numbers ranging from 65 to 996. The distribution of IBD subtypes differed across studies, with CD generally accounting for a larger proportion of cases compared with UC. The reported mean or median ages of participants spanned from 29 to 47.5 years, while the number of male participants varied substantially across studies, ranging from 36 to 468. Table 2 presents the quality assessment of the included studies.

**Table 2:** Quality assessment of included studies using NewCastle Ottawa Scale

Study (Author, Year)	Selection	Comparion	Assessment	Overall
Almuhaya et al., 2025 [17]	3	2	3	Good
Han et al., 2017 [18]	3	2	3	Good
MacMaster et al., 2021 [19]	2	1	2	Fair
Sakurai et al., 2021 [20]	3	2	2	Good
Schneider et al., 2020 [21]	4	2	2	Good
Siva et al., 2018 [12]	3	1	2	Fair
Soltani et al., 2021 [22]	3	2	3	Good

Prevalence of Zinc Deficiency

**Figure 2:**
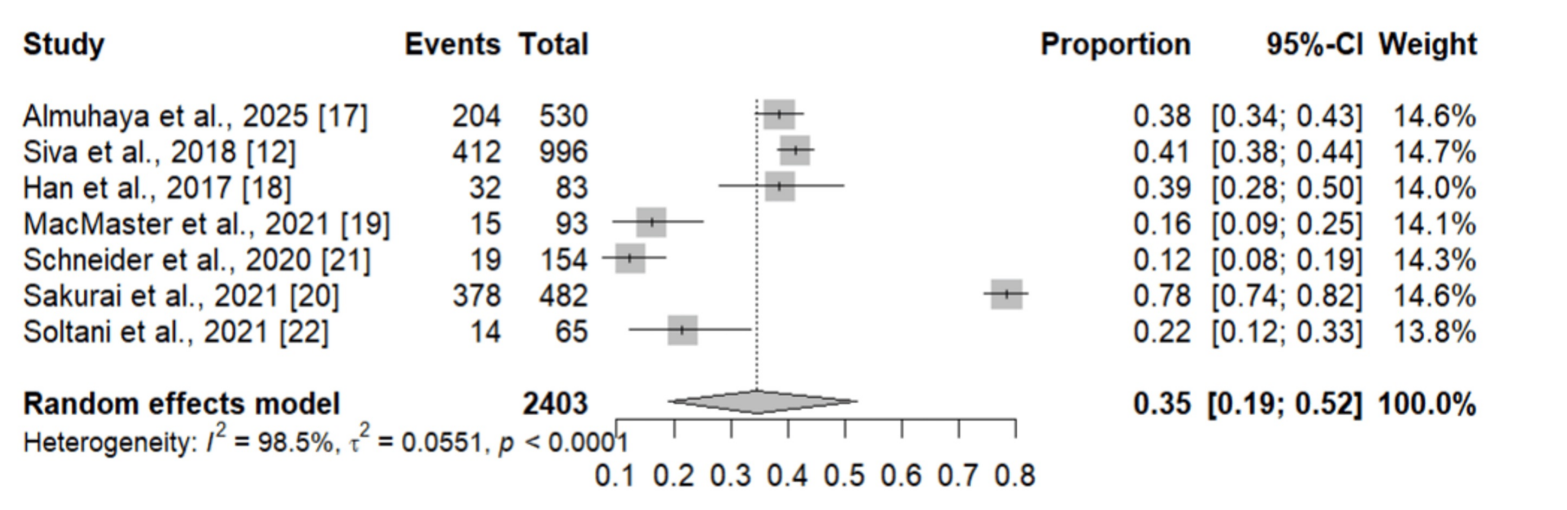
Prevalence of zinc deficiency in IBD patients. Individual study estimates are depicted as squares, with square size proportional to each study’s weight, and horizontal lines representing the corresponding 95% confidence intervals (CIs). The diamond denotes the overall pooled prevalence estimated using a random-effects model, with its width indicating the 95% CI. IBD: inflammatory bowel disease References: [12,17-22]

Figure 2 presents the pooled prevalence of zinc deficiency among patients with IBD. A total of 2,403 participants were included in the analysis, of whom 1,074 had zinc deficiency. The pooled prevalence of zinc deficiency was 35% (95% CI: 19-52%). There was substantial heterogeneity among the included studies, with an I² value of 98.5%.

**Figure 3:**
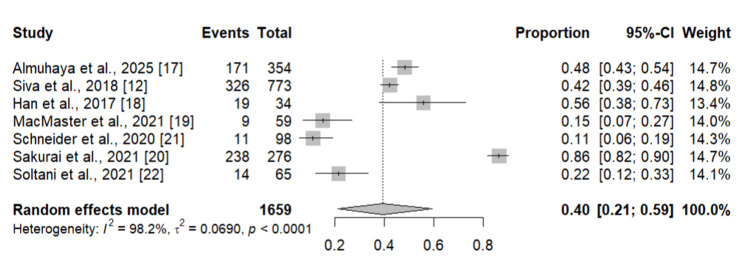
Prevalence of zinc deficiency in CD patients Individual study estimates are depicted as squares, with square size proportional to each study’s weight, and horizontal lines representing the corresponding 95% confidence intervals (CIs). The diamond denotes the overall pooled prevalence estimated using a random-effects model, with its width indicating the 95% CI. CD: Crohn’s disease References: [12,17-22]

**Figure 4:**
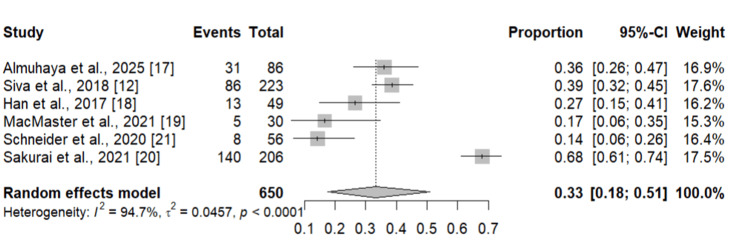
Prevalence of zinc deficiency in UC patients Individual study estimates are depicted as squares, with square size proportional to each study’s weight, and horizontal lines representing the corresponding 95% confidence intervals (CIs). The diamond denotes the overall pooled prevalence estimated using a random-effects model, with its width indicating the 95% CI. UC: ulcerative colitis References: [12,17-21]

We performed the subgroup analysis to compare the prevalence of zinc deficiency in CD and UC patients and the results are shown in Figures 3, 4, respectively. As shown in Figure 3, the pooled prevalence of zinc deficiency in CD patients was 40% (95% CI: 21-59 %). On the other hand, seven studies assessed the prevalence of zinc deficiency in UC patients. Pooled prevalence was 33% (95% CI: 18-51%).

Discussion

This systematic review and meta-analysis sought to generate an updated estimate of zinc deficiency prevalence among individuals with IBD. With increasing attention on managing chronic malabsorption conditions, zinc deficiency in both CD and UC remains an important concern. Overall, the analysis identified a pooled prevalence of zinc deficiency of 35%. When stratified by IBD subtype, the combined results showed that zinc deficiency was more common in patients with CD compared with those with UC. This finding aligns with the distinct pathological features of the two conditions: CD may involve any segment of the gastrointestinal tract, from the oral cavity to the anus, while UC is limited to the colon and rectum. Additionally, CD typically causes discontinuous, transmural inflammation that can significantly impair nutrient absorption, whereas UC is a chronic condition marked by continuous inflammation restricted to the mucosal layer of the colon [23].

The findings of this meta-analysis are consistent with, yet notably lower than, those reported in a previous systematic review and meta-analysis by Zupo et al., which estimated an overall zinc deficiency prevalence of 50% in IBD patients, with 54% in CD and 41% in UC [23]. Our study excluded studies conducted exclusively in populations with ages less than 18 years. Despite these methodological differences, both meta-analyses consistently demonstrate that zinc deficiency affects approximately one-third to one-half of IBD patients, underscoring the clinical significance of this micronutrient deficiency in this population.

Our finding that CD patients have a higher prevalence of zinc deficiency (40%) compared to UC patients (33%) is supported by physiological and anatomical considerations. Zinc is primarily absorbed in the distal duodenum and proximal jejunum. Since CD can affect any portion of the gastrointestinal tract, including these critical absorption sites, patients with CD are at substantially higher risk for zinc malabsorption [24]. A landmark study by Sturniolo et al. demonstrated that zinc absorption was considerably impaired in CD patients, ranging from 9-45%, compared with 38-75% in healthy controls [25]. Furthermore, adolescents with CD have been shown to have significantly lower zinc absorption, poorer zinc balance, and reduced plasma zinc concentrations relative to healthy children [26]. In contrast, UC primarily affects the colon and rectum, regions that play minimal roles in zinc absorption, which may explain the relatively lower, though still substantial, prevalence of zinc deficiency in this subgroup [27].

The pathogenesis of zinc deficiency in IBD is multifactorial and involves several interconnected mechanisms. First, reduced dietary intake due to disease-related symptoms such as nausea, abdominal pain, anorexia, and self-imposed dietary restrictions to control symptoms contributes significantly to inadequate zinc consumption [11]. Second, active intestinal inflammation disrupts the integrity of the intestinal mucosal barrier and impairs the absorption of nutrients, including zinc [28]. The inflammatory process itself increases intestinal zinc losses through diarrhea, a hallmark symptom of IBD, which further exacerbates deficiency. Additionally, patients with CD who have undergone small bowel resections experience reduced absorptive surface area, compounding the risk of zinc deficiency [29].

The substantial heterogeneity observed in this meta-analysis (I² = 98.5%) deserves careful consideration. Several factors contribute to this heterogeneity. First, there is considerable variability in the definition of zinc deficiency across studies, with cut-off values ranging from <0.66 μg/mL to <10.7 μmol/L [23]. This lack of standardization makes direct comparisons challenging and may inflate or deflate prevalence estimates depending on the threshold used. Second, studies included populations with varying disease characteristics, including differences in disease duration, activity status, anatomical distribution, and treatment regimens, all of which may influence zinc status.

The results of this meta-analysis carry several important clinical implications. First, they highlight the need for increased awareness among clinicians regarding the high prevalence of zinc deficiency in IBD patients, particularly those with CD. Routine screening for zinc deficiency should be considered as part of the comprehensive nutritional assessment of IBD patients, especially in those with risk factors such as extensive small bowel disease, previous resections, chronic diarrhea, or malnutrition [30]. Early identification and correction of zinc deficiency may help prevent adverse clinical outcomes and improve overall disease management. Second, given the association between zinc deficiency and poor clinical outcomes, as well as the improvement in outcomes following zinc normalization, clinicians should prioritize zinc repletion in deficient patients. While the optimal approach to zinc supplementation in IBD requires further study, available evidence suggests that oral zinc supplementation at doses ranging from 35-110 mg of elemental zinc per day is generally safe and may provide clinical benefits [8]. Careful monitoring of zinc levels during supplementation is important to avoid excess, as zinc supplementation at very high doses (>40 mg/day long-term) can interfere with copper absorption and immune function [31].

Limitations

The findings should be interpreted with caution due to the relatively small number of studies meeting the inclusion criteria (n = 7), which limits statistical power and the ability to conduct extensive subgroup analyses. The retrospective nature of most included studies introduces potential for selection bias and residual confounding. Furthermore, we were unable to assess the impact of specific disease phenotypes (such as disease location in CD), disease activity status, or concurrent medications on zinc deficiency prevalence due to insufficient data in the primary studies. Future prospective studies with standardized zinc assessment methods and comprehensive phenotypic characterization are needed to address these gaps.

Future Directions

From a research perspective, several critical questions remain unanswered and should be prioritized for future investigation. First, large-scale randomized controlled trials are required to establish whether zinc supplementation improves clinical outcomes in IBD patients, including disease activity scores, mucosal healing, quality of life, and rates of hospitalization and surgery. Second, studies should investigate optimal dosing regimens, formulations (e.g., zinc sulfate vs. zinc gluconate vs. zinc carnosine), and duration of therapy. Third, research should focus on identifying which subgroups of IBD patients are most likely to benefit from zinc supplementation, potentially through biomarker-guided approaches. Fourth, mechanistic studies are needed to further elucidate the molecular pathways through which zinc influences intestinal inflammation, barrier function, immune regulation, and the gut microbiome. Finally, efforts should be made to develop and validate more accurate and comprehensive methods for assessing zinc status that overcome the limitations of serum zinc measurements.

## Conclusions

This systematic review and meta-analysis demonstrates that zinc deficiency is highly prevalent in patients with IBD, affecting approximately one-third of the overall IBD population. The prevalence observed in patients with CD was higher compared to UC, which reflects the distinct pathophysiological mechanisms and anatomical involvement patterns of these conditions. These findings emphasize the critical importance of routine zinc status assessment in IBD management, particularly for patients with CD, extensive small bowel involvement, or chronic malabsorption. Early identification and appropriate supplementation of zinc deficiency may improve clinical outcomes, enhance quality of life, and optimize therapeutic responses. Future research should focus on standardizing zinc assessment methods, establishing evidence-based supplementation protocols, and evaluating the impact of zinc repletion on disease activity, mucosal healing, and long-term patient outcomes through well-designed randomized controlled trials.
